# Baseline markers and exploratory analysis of post-treatment thyroid autoantibody seroconversion for immune-related thyroid dysfunction in patients with advanced non-small-cell lung cancer treated with ICIs: a prospective single-center cohort study

**DOI:** 10.3389/fonc.2026.1975574

**Published:** 2026-10-20

**Authors:** Hui Ming, Qun Liang, Wenbing Hu, Yuanhao Chen, Hui Yu

**Affiliations:** 1 Department of Nuclear Medicine, Huangshi Central Hospital, Affiliated Hospital of Hubei Polytechnic University, Huangshi, China; 2 Office of Medical Research, Huangshi Central Hospital, Affiliated Hospital of Hubei Polytechnic University, Huangshi, China; 3 Department of Oncology, Huangshi Central Hospital, Affiliated Hospital of Hubei Polytechnic University, Huangshi, China

**Keywords:** immune checkpoint inhibitors, Immune-related adverse events, non-small-cell lung cancer, Thyroglobulin antibody, Thyroid peroxidase antibody

## Abstract

**Objective:**

To evaluate the predictive value of baseline thyroid peroxidase antibodies (TPOAb), thyroglobulin antibodies (TgAb), and post-treatment seroconversion for thyroid immune-related adverse events (irAEs) in patients with advanced non-small cell lung cancer (NSCLC) receiving immune checkpoint inhibitors (ICIs).

**Methods:**

Treatment-naive patients with advanced NSCLC who received ICIs between January 2021 and June 2026 were prospectively enrolled. Patients with pre-existing thyroid disorders, other autoimmune diseases, or baseline use of immunosuppressive-dose glucocorticoids/immunosuppressants were excluded. Thyroid function, TPOAb, and TgAb were measured prior to ICI initiation. Competing risk analysis was applied to assess the cumulative incidence of thyroid irAEs, with death treated as the competing event. Cox regression assessed baseline antibody status, baseline TSH, and seroconversion (as a time-dependent covariate) in relation to irAE risk. The proportional hazards assumption was assessed, and a stratified Cox model with a time-dependent covariate was used when violated.

**Results:**

A total of 312 patients with complete follow-up data were included in the final analysis. The median follow-up duration was 6.4 months. Baseline TPOAb and TgAb positive rates were 18.9% and 14.1%. The overall incidence of thyroid-related irAEs was 27.9% (87/312). Positive baseline TPOAb and TgAb were associated with higher irAE incidence (57.6% vs. 20.9% and 61.4% vs. 22.4%, both *P* < 0.001). In the competing-risk setting accounting for death as a competing event, patients with positive baseline thyroid antibodies had a significantly greater cumulative incidence of thyroid irAEs during follow-up (sHR = 3.353, *P* < 0.001). In the final time-dependent model, baseline antibody positivity was significantly associated with irAEs at baseline (HR = 17.529, *P* < 0.001), with a significant time interaction (*P* < 0.001). Baseline TSH remained an independent predictor (HR = 2.471, 95% CI: 1.845-3.309, *P* < 0.001). In the crude analysis, seroconversion was associated with a higher incidence of thyroid irAEs. However, in the time-dependent Cox model restricted to baseline double-negative patients, seroconversion was not an independent predictor of thyroid irAEs (HR = 0.236, 95% CI: 0.05-1.041, *P* = 0.056). The area under the receiver operating characteristic (ROC) curve for baseline TSH was 0.818, with an exploratory cut-off value of 2.985 mIU/L.

**Conclusion:**

In this single-center exploratory cohort, baseline TPOAb/TgAb positivity and elevated TSH were associated with thyroid irAEs after ICIs. These findings are hypothesis-generating and require external validation. The TSH cut-off (2.985 mIU/L) is exploratory and not clinically actionable. Post-treatment antibody seroconversion was not an independent predictor and remains exploratory. Serial monitoring may aid risk stratification but needs validation.

## 1 Introduction

Immune checkpoint inhibitors (ICIs) have substantially improved clinical outcomes in advanced non-small cell lung cancer (NSCLC). However, the overactivation of the immune system can provoke immune-related adverse events (irAEs), among which thyroid immune-related adverse events (irTD) are the most common endocrine toxicity (1). irTD present with heterogeneous clinical manifestations, including subclinical thyrotoxicosis, destructive thyrotoxicosis, and hypothyroidism. Some patients develop permanent thyroid damage requiring long-term hormone replacement, which may interfere with anti-tumor therapy and reduce quality of life (2). Reported irTD incidence varies widely across studies, and identifying high-risk patients before immunotherapy initiation remains a pressing clinical challenge (3).

Baseline thyroid autoantibodies (TPOAb and TgAb) are the most extensively investigated potential biomarkers for irTD prediction. Multiple cohort studies have confirmed that tumor patients with positive baseline thyroid autoantibodies are at significantly increased risk of irTD after ICI therapy (4). Nevertheless, existing evidence indicates that while baseline thyroid autoantibodies have high specificity, their sensitivity is limited; a considerable proportion of irTD patients are negative at baseline, suggesting alternative pathogenic mechanisms independent of pre-existing humoral autoimmunity (5).

Beyond autoantibodies, even within the normal laboratory reference range, relatively higher baseline TSH levels have been associated with increased irTD risk, possibly reflecting thyroid reserve capacity and subclinical immune status (6). Recently, some studies have moved beyond static baseline measurements to investigate dynamic changes in thyroid autoantibodies during immunotherapy. Treatment-emergent antibody titer increases or seroconversion may provide additional information for irTD risk stratification. However, most current evidence comes from retrospective studies and lacks prospective validation (7).

Current international guidelines do not universally recommend routine baseline thyroid autoantibody screening before ICI therapy, primarily due to insufficient high-quality prospective real-world data, particularly in Chinese populations. Most previous studies have been retrospective, and few have simultaneously evaluated the predictive value of baseline antibodies, post-treatment seroconversion, and baseline TSH. Against this backdrop, we conducted a single-center prospective cohort study in advanced NSCLC patients to investigate the predictive role of baseline thyroid autoantibodies, post-treatment seroconversion, and baseline TSH in irTD, aiming to inform risk stratification and monitoring strategies in clinical practice.

## 2 Materials and methods

### 2.1 Study design and participants

This single-center prospective cohort study consecutively enrolled treatment-naϊve patients with advanced NSCLC at Huangshi Central Hospital from January 2021 to June 2026. Inclusion criteria were (1): histologically or cytologically confirmed stage IIIB–IV NSCLC (2); met indications for ICI therapy and planned to receive ICI monotherapy, dual ICI combination, or ICI combined with other conventional anti-tumor regimens (3); age ≥18 years (4); Eastern Cooperative Oncology Group (ECOG) performance status 0–2 (5); expected survival ≥3 months and ability to undergo regular follow-up with thyroid function assessments (6); normal baseline thyroid function.

Exclusion criteria were (1): current or prior thyroid disease (including hyperthyroidism, hypothyroidism, Hashimoto’s thyroiditis, Graves’ disease, etc.) (2); other autoimmune diseases (3); baseline use of equivalent prednisone ≥10 mg/day glucocorticoids or immunosuppressants (short-term low-dose glucocorticoids for antiemesis during chemotherapy were permitted) (4); prior ICI therapy (5); pregnancy or lactation (6); incomplete clinical data.

### 2.2 Treatment regimens

All enrolled patients received ICI therapy, including programmed death protein-1 (PD-1) inhibitors (e.g., nivolumab, pembrolizumab, sintilimab) or programmed death-ligand 1 (PD-L1) inhibitors (e.g., atezolizumab). Regimens comprised ICI monotherapy, dual ICI combination, or ICI combined with other conventional anti-tumor therapies, administered according to the package inserts (every 2–4 weeks).

### 2.3 Thyroid function and autoantibody assays

Peripheral venous blood was collected one day prior to the first ICI infusion. Thyroid-stimulating hormone (TSH), free triiodothyronine (FT3), free thyroxine (FT4), TPOAb, and TgAb were quantified via chemiluminescent immunoassay. Assay cut-offs: TPOAb > 40 IU/mL, TgAb > 95 IU/mL according to manufacturer specifications. Thyroid function panels plus autoantibodies were repeated every six weeks. Post-treatment seroconversion was defined as values exceeding assay thresholds in patients who were TPOAb/TgAb-negative at baseline.

### 2.4 Definition and assessment of thyroid-related irAEs

Thyroid-related irAEs were defined based on biochemical criteria consistent with established diagnostic frameworks for endocrine immune-related adverse events (8), and included the following categories (1): hypothyroidism: elevated TSH with decreased FT4, or TSH ≥10 mIU/L requiring levothyroxine replacement (2); hyperthyroidism: decreased TSH with elevated FT3/FT4 (3); subclinical thyroid dysfunction: abnormal TSH with normal FT3 and FT4 levels. All abnormal thyroid function results were reviewed by an endocrinologist to exclude other etiologies (e.g., non-thyroidal illness syndrome, medications, other underlying conditions) before confirming a thyroid irAE diagnosis. Antibody seroconversion was defined as TPOAb or TgAb exceeding the upper reference limit at any follow-up visit in patients who were negative at baseline.

### 2.5 Follow-up

Patients were followed from the date of first ICI administration, with thyroid function and autoantibody assessments scheduled every 6 weeks (allowable window ±2 weeks). Follow-up endpoints were occurrence of thyroid irAEs, ICI therapy discontinuation, death, or the study cut-off date. A total of 312 patients with complete follow-up data were included in the final analysis. Patients who withdrew, were lost to follow-up, transferred, or had incomplete data after enrollment were excluded from the analysis.

### 2.6 Statistical analysis

Statistical analyses were performed using SPSS version 19.0. Categorical variables were presented as counts (percentages) and compared using Pearson’s χ² test, or Fisher’s exact test when expected frequencies were insufficient. Non-normally distributed continuous variables were expressed as median (interquartile range) and compared using the Mann–Whitney U test. Competing-risk analysis was performed using the cmprsk package in R. The cumulative incidence function (CIF) was estimated using the Aalen-Johansen estimator, and Gray’s test was used for group comparisons. Univariate and multivariable Cox proportional hazards models were used to estimate hazard ratios (HRs) and 95% confidence intervals (CIs) for associations with thyroid irAE risk. For categorical variables, the reference categories were baseline antibody-negative and non-seroconversion. Univariate Cox regression was used for variable screening, and variables with *P* < 0.2 were considered for inclusion in the multivariable model. The proportional hazards assumption was assessed using Schoenfeld residuals and time-dependent interaction terms. When the assumption was violated *(P* < 0.05), a stratified Cox regression model or a time-dependent covariate was applied, as appropriate. Collinearity was assessed using variance inflation factors (VIF). Receiver operating characteristic (ROC) curve analysis was performed to evaluate the predictive performance of baseline TSH for thyroid irAEs; the optimal cut-off was determined by maximizing the Youden index, and the area under the curve (AUC), sensitivity, and specificity were calculated. A two-sided *P* < 0.05 was considered statistically significant.

## 3 Results

### 3.1 Baseline clinical characteristics

A total of 339 patients were initially enrolled and followed prospectively. During follow-up, 27 patients were excluded: 14 withdrew voluntarily, 6 were lost to follow-up, 5 transferred to another hospital, and 2 had incomplete key data. Finally, 312 eligible advanced NSCLC patients with complete follow-up data were included in the analysis (**Figure 1**), including 271 males (86.8%) and 41 females (13.2%). Median age was 63 (58–69) years; median BMI was 21.51 (19.13–23.74) kg/m²; median baseline TSH was 2.57 (2.11–3.29) μIU/mL; median follow-up duration was 192 (84–421) days. Histological subtypes were adenocarcinoma in 154 (49.4%), squamous cell carcinoma in 108 (34.6%), large cell carcinoma in 40 (12.8%), and other types in 10 (3.2%). A history of smoking was present in 208 patients (66.7%). ICI regimens included PD-1 inhibitors in 277 (88.8%), PD-L1 inhibitors in 25 (8.0%), and ICI combination therapy in 10 (3.2%). Baseline TPOAb positivity was observed in 59 patients (18.9%), and TgAb positivity in 44 (14.1%).

**Figure 1:**
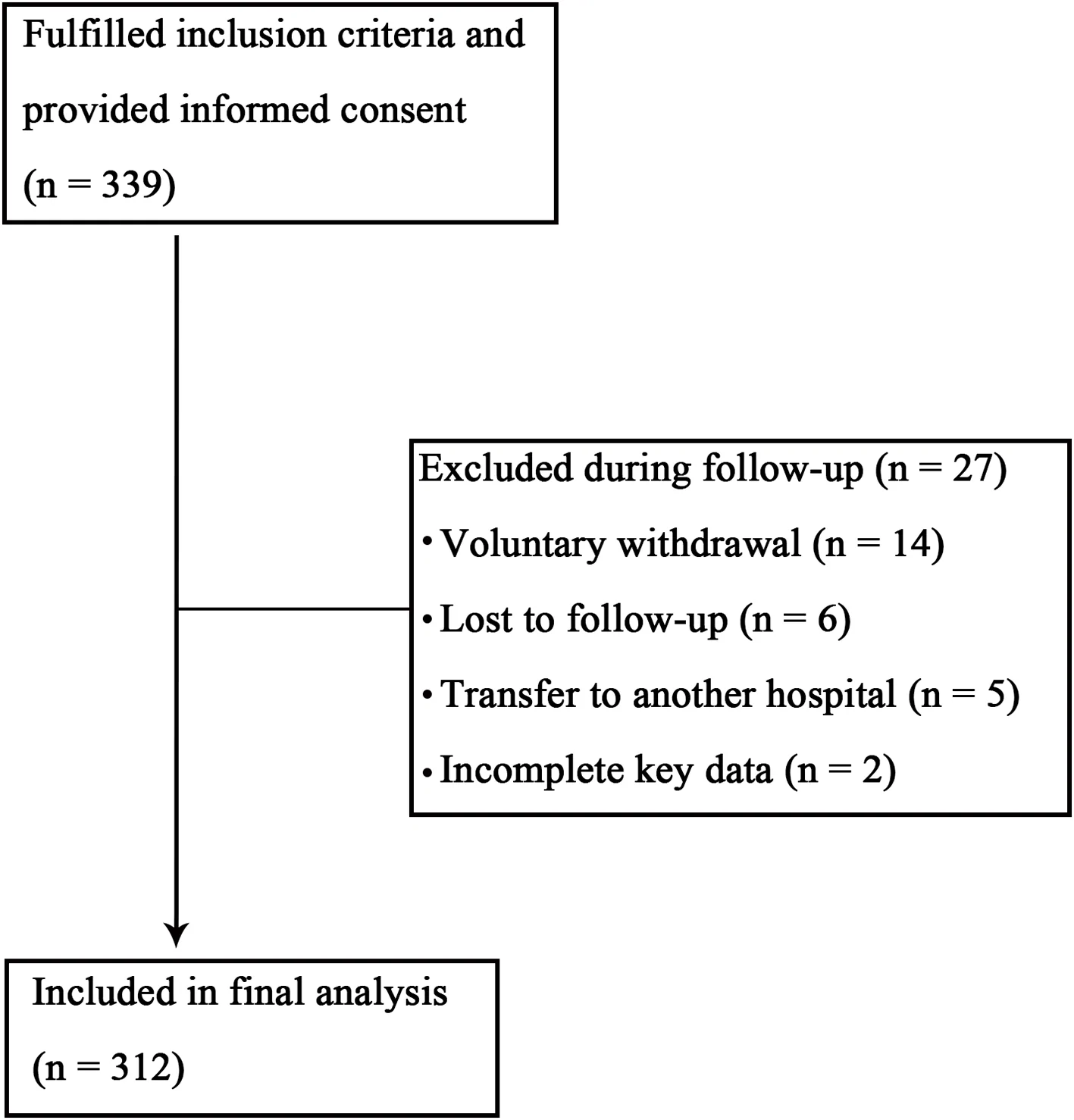
Patient flow diagram .

Among the 87 patients with thyroid irAEs, 62 (71.3%) had hypothyroidism, 13 (14.9%) had hyperthyroidism, and 12 (13.8%) had subclinical thyroid dysfunction. The median time to onset was 12 weeks for hypothyroidism, 6 weeks for hyperthyroidism, and 15 weeks for subclinical dysfunction.

Patients were stratified by baseline thyroid autoantibody status into baseline-negative (n=234) and baseline-positive (n=78) groups. Baseline characteristics are summarized in **Table 1**. No significant differences were observed between the two groups in age, sex, histological type, smoking history, tumor stage, ICI regimen, or number of ICI cycles (all *P*>0.05), indicating comparability. However, significant differences were found in baseline TSH (Z=-5.102, *P* < 0.001) and incidence of thyroid irAEs (χ²=34.858, *P* < 0.001).

**Table 1:** Comparison of clinical baseline characteristics between patients with baseline negative and positive thyroid autoantibodies.

Characteristics	Baseline antibody negative(n=234)	Baseline antibody positive(n=78)	*P* value
Sex, n			0.208
Male	200	71	
Female	34	7	
Age	63 (58-69)	66 (59-71)	0.1
BMI	21.50 (19.15-23.56)	21.54 (18.61-24.11)	0.624
Baseline TSH	2.47 (1.98-3.01)	3.22 (2.41-3.67)	<0.001
Smoking history, n			0.782
Yes	155	53	
No	79	25	
Pathological type, n			0.912
Adenocarcinoma	118	36	
Squamous cell carcinoma	80	28	
Large-cell carcinoma	29	11	
Others	7	3	
Tumor stage, n			0.705
Stage IIIB/IIIC	59	18	
Stage IV	175	60	
ICI regimen, n			0.377
PD-1 inhibitor	211	66	
PD-L1 inhibitor	16	9	
Combination therapy	7	3	
Number of ICI cycles	6(3-14)	7(4-14)	0.517
Follow-up time (days)	173(84-423)	221(101-419)	0.356
Thyroid-related irAEs, n			<0.001
Yes	45	42	
No	189	36	

### 3.2 Association between baseline TPOAb/TgAb and thyroid-related irAEs

The crude incidence of thyroid irAEs according to baseline antibody status was compared using 2×2 contingency tables (**Table 2**). Both baseline TPOAb-positive and TgAb-positive groups had significantly higher crude incidence rates compared to their respective negative groups (*P* < 0.001). When stratified into three subgroups (double-negative, single-positive, double-positive), intergroup differences in thyroid irAE incidence were also significant (*P* < 0.001). Competing risk analysis using the CIF demonstrated that the baseline-positive group had a significantly higher cumulative incidence of thyroid irAEs throughout the entire follow-up period compared to the baseline-negative group (Gray test χ² = 36.687, *P* < 0.001, **Figure 2**). In univariate Fine-Gray regression, baseline antibody positivity was associated with an increased subdistribution hazard of thyroid irAEs (sHR=3.353, 95%CI: 2.256-4.985, *P* < 0.001).

**Table 2:** Baseline TPOAb and TgAb status in patients with and without thyroid-related irAEs.

Characteristics	Thyroid-related irAEs (n=87)	No thyroid-related irAEs (n=225)	χ²	*P* value
Baseline TgAb, n			28.551	<0.001
Negative	22.4% (60)	77.6% (208)		
Positive	61.4% (27)	38.6% (17)		
Baseline TPOAb, n			23.007	<0.001
Negative	20.9% (53)	79.1% (200)		
Positive	57.6% (34)	42.2% (25)		
Baseline autoantibody status, n			43.838	<0.001
Double-negative	19.2% (45)	80.8% (189)		
Double-positive	76.0% (19)	24.0% (6)		
Single-positive	43.4% (23)	56.6% (30)		

**Figure 2:**
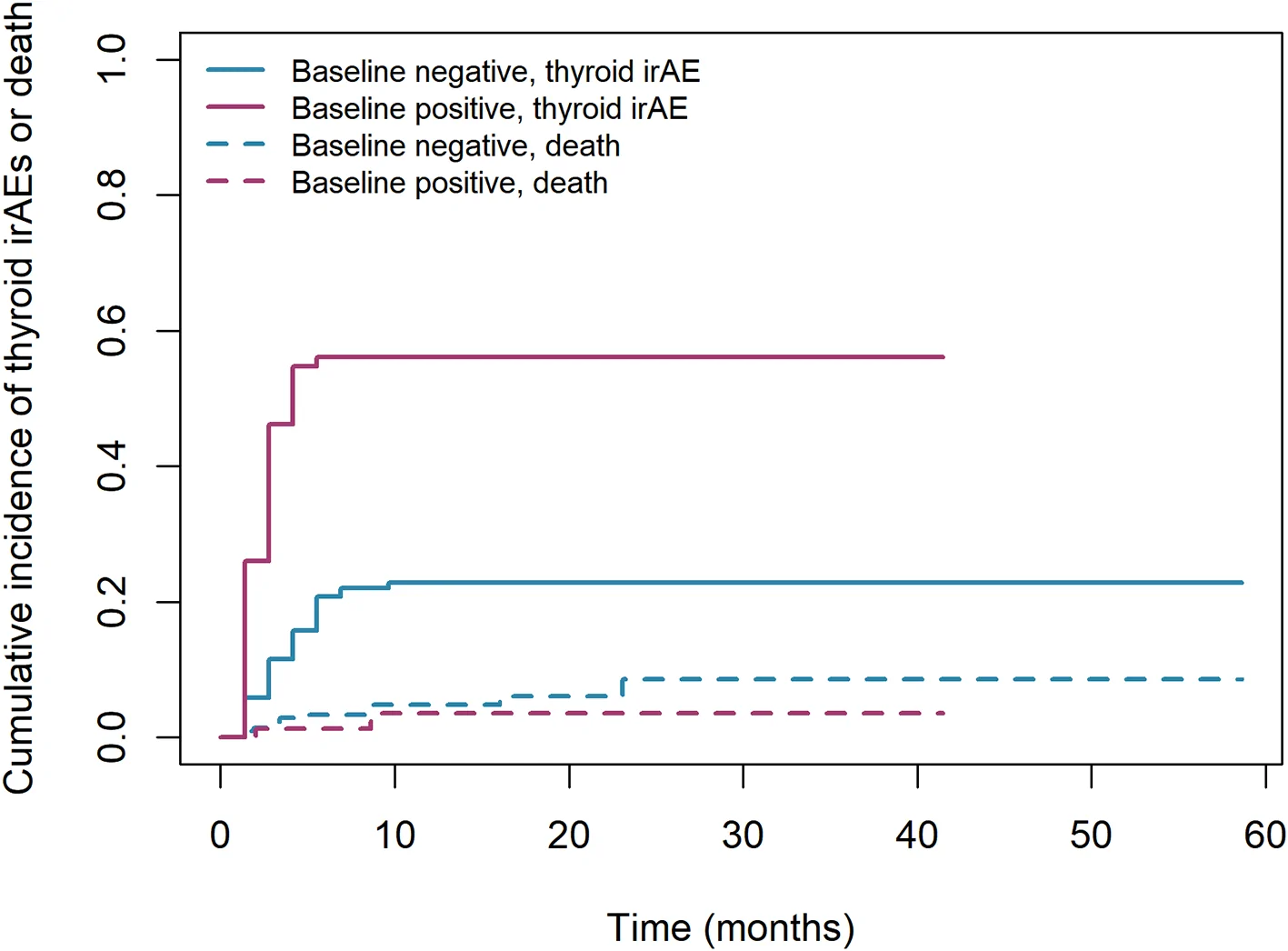
Cumulative incidence curves for thyroid-related irAEs stratified by baseline thyroid antibody status.

### 3.3 Cox regression analysis for thyroid irAE risk

Univariate Cox regression was first performed for all clinical and laboratory variables to identify factors associated with thyroid irAEs after ICI therapy. Variables with *P* < 0.2 in univariate analysis (BMI, tumor stage, baseline antibody positivity, baseline TSH) were entered into the initial multivariable Cox model (9, 10). The proportional hazards assumption was tested using Schoenfeld residuals and time-dependent interaction terms. The assumption was violated for tumor stage and baseline antibody positivity (both *P* < 0.05). Therefore, a stratified Cox regression model was used with tumor stage as the stratification factor, and a time-dependent covariate (follow-up time×baseline antibody positivity) was included for antibody positivity. Collinearity diagnostics showed all VIF values <5, and the events per variable was 21.75 (87 events/4 variables).

In the final model, baseline antibody positivity was a significant predictor at baseline (HR = 17.529, 95% CI: 7.339-41.866, *P* < 0.001). The time interaction term was significant (*P* < 0.001), indicating that the effect of antibody positivity decreased over time. Baseline TSH remained an independent predictor (HR = 2.471, 95% CI: 1.845-3.309, *P* < 0.001). BMI was not an independent predictor (HR = 1.017, 95% CI: 0.957-1.080, *P* = 0.586).

### 3.4 ROC curve analysis for baseline TSH

ROC curve analysis demonstrated that baseline TSH was associated with thyroid irAEs throughout the entire follow-up period, with an AUC of 0.818 (95% CI: 0.763–0.873, *P* < 0.001). The exploratory cut-off value determined by the Youden index was 2.985 mIU/L, with a corresponding sensitivity of 71.3% and specificity of 81.3% (**Figure 3**). Based on this cut-off, the positive predictive value (PPV) was 59.6% (95% CI: 50.0–68.7%) and the negative predictive value (NPV) was 88.0% (95% CI: 82.9–91.6%). When baseline TSH was dichotomized at this cut-off and included in the Cox model, the dichotomized baseline TSH remained an independent risk factor for thyroid irAEs after adjusting for confounders (BMI, tumor stage, baseline antibody positivity) (HR = 4.304, 95% CI: 2.644–7.004, *P* < 0.001), indicating robustness. However, this threshold is exploratory and internally derived, and requires external validation before clinical use.

**Figure 3:**
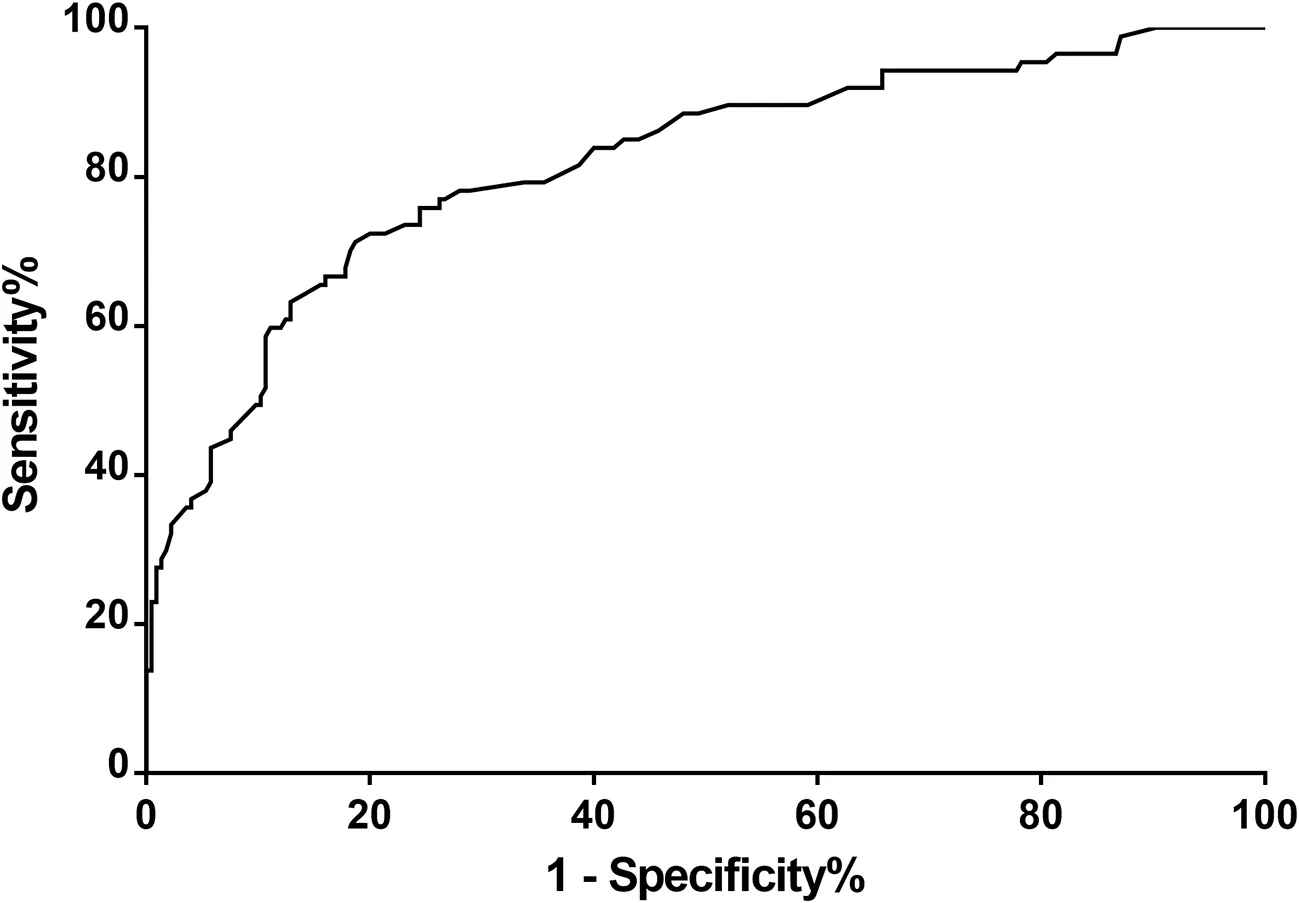
ROC curve of baseline TSH for predicting the development of thyroid-related irAEs.

### 3.5 Post-treatment autoantibody seroconversion

In patients with negative baseline TPOAb, the TPOAb seroconversion rate was 12.6% (32/253); in patients with negative baseline TgAb, the TgAb seroconversion rate was 9.7% (26/268). The median time to seroconversion was 12 weeks. Among the 234 patients who were double-negative at baseline, 36 (15.4%) developed seroconversion for at least one antibody; the remaining 198 (84.6%) did not seroconvert. Among the 36 patients who seroconverted, 22 also developed thyroid irAEs. In these 22 patients, irAE onset preceded seroconversion in 9 (40.9%), seroconversion preceded irAE onset in 6 (27.3%), and both occurred on the same day in 7 (31.8%).

In the crude analysis, seroconversion was associated with a significantly higher incidence of thyroid irAEs. Among baseline TgAb-negative patients, the irAE incidence was 57.7% (15/26) in those who seroconverted versus 18.6% (45/242) in those who did not (χ²=20.654, *P* < 0.001). Among baseline TPOAb-negative patients, the irAE incidence was 62.5% (20/32) in those who seroconverted versus 14.9% (33/221) in those who did not (χ²=38.193, *P* < 0.001).

Because seroconversion occurred during follow-up rather than at baseline, it was treated as a time-dependent covariate in Cox regression analysis restricted to the baseline double-negative subgroup. With seroconversion as the exposure group and non-seroconversion as the reference group, post-treatment seroconversion was not an independent predictor for thyroid irAEs after adjusting for confounders (HR = 0.236, 95% CI: 0.053-1.041, *P* = 0.056). A landmark analysis at 12 weeks was performed to mitigate immortal time bias. In this analysis, seroconversion by 12 weeks was not significantly associated with subsequent thyroid irAEs (HR = 0.822, 95% CI: 0.351-1.927, *P* = 0.653).

## 4 Discussion

The interindividual risk of immune checkpoint inhibitor-related thyroid immune damage (irTD) varies considerably. Existing evidence suggests that both baseline thyroid autoimmunity and thyroid reserve function contribute to disease development; however, the effect sizes and predictive performance of reported biomarkers are not entirely consistent across cohorts (11). Multiple original cohorts and meta-analyses have confirmed that baseline TPOAb and TgAb positivity are associated with increased irTD risk, but these markers generally exhibit high specificity with limited sensitivity—a substantial proportion of irTD patients are baseline-negative for thyroid autoantibodies, suggesting alternative pathogenic pathways independent of pre-existing humoral autoimmunity (5, 11). Most previous studies have focused on static baseline measurements, but recent work has begun to examine dynamic changes in thyroid autoantibodies during immunotherapy. Some retrospective data suggest that early on-treatment antibody changes may outperform baseline testing for irTD prediction, though these findings lack prospective external validation (7). Meanwhile, even within the laboratory reference range, relatively higher baseline TSH is also considered a risk factor for irTD, reflecting subclinical thyroid injury and diminished reserve capacity; however, optimal warning thresholds vary across studies due to differences in assay platforms and population characteristics (6, 12). Furthermore, most available evidence derives from retrospective studies, and data from one tumor type cannot be directly extrapolated to others. For example, Wang Z et al. demonstrated predictive value of thyroid axis markers in a gastric cancer neoadjuvant immunochemotherapy cohort, but that study focused on perioperative gastric cancer patients, a clinical setting distinctly different from palliative immunotherapy for advanced NSCLC, limiting generalizability (13). Against this background, our prospective cohort simultaneously evaluated baseline autoantibodies, baseline TSH, and post-treatment seroconversion in advanced NSCLC to provide real-world data for irTD risk stratification in a Chinese population.

Our finding that baseline thyroid autoantibody positivity is an independent risk factor for irTD aligns with most published cohort studies (3, 14, 15). From a pathophysiological perspective, baseline TPOAb/TgAb positivity often indicates subclinical autoimmune changes, including lymphocytic infiltration within thyroid follicles. When the PD-1/PD-L1 immunosuppressive pathway is blocked, autoreactive T cells are massively activated and further attack thyroid tissue, ultimately triggering irTD (16). However, we also observed that some irTD patients were baseline-negative for thyroid autoantibodies. Consistent with Muir et al., baseline thyroid autoantibodies, while specific, have sensitivity of only 20–31%, suggesting that CD8^+^ T cell-mediated direct thyroid follicular destruction can occur without pre-existing humoral thyroid autoimmunity, and baseline antibody screening alone cannot identify all high-risk individuals (5). Risk effect estimates vary considerably across studies; Meta-analysis indicated that population ethnicity, tumor composition, follow-up frequency, and assay reagents all contribute to between-study heterogeneity (11). Notably, Jiao et al. (2025) in a multi-tumor retrospective cohort reported that only baseline TPOAb and TRAb were independent risk factors for irTD, while TgAb showed no independent predictive role, suggesting that the contribution of different autoantibodies to irTD pathogenesis may vary across cohorts, possibly due to differences in tumor spectrum, antibody assay kits, and irTD subtype distribution (17).

In baseline-negative patients, we treated post-treatment seroconversion as a time-dependent covariate to avoid time bias. The association between seroconversion and irTD risk did not reach statistical significance (*P* = 0.056). Several explanations may account for this finding. First, after thyroid follicles are destroyed by immune attack, abundant thyroid antigens are released, stimulating B-cell activation and autoantibody production; seroconversion may be a secondary event following thyroid tissue injury rather than an antecedent driver of irTD (18). Second, available literature indicates that post-treatment antibody titer increases or seroconversion are more concentrated in the clinically overt destructive thyrotoxicosis subgroup and are relatively less common in isolated hypothyroidism or subclinical dysfunction. Our cohort included multiple clinical subtypes of irTD, and phenotypic heterogeneity may have diluted the association (2). Finally, the number of cases with both seroconversion and irTD in the baseline-negative subgroup was limited, raising the possibility of a type II statistical error. Taken together, current evidence does not support post-treatment seroconversion as an independent predictor of irTD, and clinical intervention based solely on newly emergent antibody positivity during follow-up is not recommended. The potential clinical utility of post-treatment seroconversion remains uncertain and requires further validation in larger prospective cohorts stratified by irTD clinical phenotype.

Our findings reaffirm that risk assessment should not rely solely on whether TSH exceeds the laboratory reference range; the relative level of baseline TSH within the normal range also carries predictive significance. The exploratory TSH cut-off in our cohort was 2.985 mIU/L, with an AUC of 0.818 from a standard ROC analysis. However, because this analysis was applied to a time-to-event outcome with variable follow-up, the threshold should be considered exploratory and internally derived, and external validation is required before clinical use. The PPV was 59.6% (95% CI: 50.0–68.7%) and the NPV was 88.0% (95% CI: 82.9–91.6%), indicating that this threshold may be more useful for ruling out patients at lower risk rather than definitively identifying those who will develop irAEs. Previous cohorts have similarly found that mildly elevated baseline TSH is associated with increased irTD risk, although the specific warning thresholds vary across studies (6, 12). Interestingly, baseline TSH did not emerge as an independent risk factor in the retrospective cohort of Jiao Y et al., further suggesting that the predictive performance of baseline TSH is influenced by study population, tumor composition, and irTD subtype distribution, and that cut-offs from a single cohort should not be universally applied (17). Mildly elevated baseline TSH may reflect diminished thyroid reserve and subclinical autoimmune status; when challenged by ICI-mediated immune attack, the compensatory capacity is insufficient, predisposing to thyroid dysfunction. Combining baseline TSH with thyroid autoantibodies may help identify high-risk patients, consistent with the variable selection strategy of previously published irTD prediction nomograms (19). However, this threshold requires validation in larger cohorts before routine clinical use, and clinical decisions should not rely solely on this cut-off (2.985 mIU/L).

ICI-induced thyroid injury exhibits substantial clinical heterogeneity. Some patients experience a biphasic “thyrotoxicosis–hypothyroidism” course characteristic of destructive thyroiditis, while others present directly with hypothyroidism without a thyrotoxic phase; subclinical dysfunction is also common (2, 18). Most cohorts have observed that TRAb is negative in the majority of irTD cases, indicating that Graves’ disease is not the primary etiology; the vast majority of irTD represents T cell-mediated destructive thyroiditis (4). Due to limited sample size, we did not perform stratified analyses by irTD subtype; the distribution of baseline and dynamic thyroid autoantibodies across different phenotypes requires further investigation.

Some retrospective cohorts have observed survival benefits in tumor patients who develop irTD. The prevailing hypothesis favors a mediation effect: pre-existing thyroid autoimmunity increases irTD risk, and the occurrence of irTD indicates activation of anti-tumor immunity—rather than direct improvement of anti-tumor efficacy by thyroid autoantibodies themselves. Moreover, this survival association may be confounded by factors such as immunotherapy line and tumor type (20, 21). It should be noted that our study’s primary endpoint was limited to thyroid immune-related adverse events; we did not design survival or anti-tumor efficacy endpoints, and therefore we do not draw inferences regarding relationships between antibodies, irTD, and tumor prognosis, to avoid overextrapolation.

Several limitations of this study should be acknowledged. As a single-center prospective cohort, all participants had advanced NSCLC, and generalizability to other tumor types such as gastric cancer or melanoma requires caution. Although 27 of 339 enrolled patients were excluded from the final analysis due to withdrawal, loss to follow-up, transfer, or incomplete data, these reasons were largely unrelated to thyroid irAE risk. This may introduce some selection bias, but its impact on the main findings is likely limited. Although prospective in design, the sample size was limited, and the number of events with both post-treatment seroconversion and irTD in the baseline-negative subgroup was insufficient, reducing statistical power for this analysis. We did not measure TRAb, preventing complete differentiation of the small subset of ICI-induced Graves’-like thyrotoxicosis. The positivity cut-offs for TPOAb, TgAb, and TSH were derived from our institution’s assay kits; reference ranges may differ across laboratories and institutions, and the TSH cut-off obtained here should not be directly applied to other clinical centers. Our study focused solely on thyroid toxicity endpoints and did not collect tumor survival or efficacy data, nor did we include routine blood counts or inflammatory markers; we also did not perform external validation of previously published irTD prediction nomograms.

### 5 Conclusions

In this single-center exploratory cohort, baseline TPOAb/TgAb positivity and elevated baseline TSH were associated with thyroid irAEs in advanced NSCLC patients treated with ICIs. However, these findings are hypothesis-generating and require external validation. A TSH cut-off of 2.985 mIU/L is exploratory and should not be used as a clinically actionable threshold. Baseline autoantibody negativity does not exclude irAE risk, and regular follow-up is still needed. Post-treatment seroconversion was not an independent predictor in the time-dependent Cox model and should be considered exploratory; it should not be used as a sole trigger for intervention. Integrating baseline thyroid parameters with dynamic monitoring may support individualized risk stratification and follow-up, but further validation is needed.

## Data Availability

The original contributions presented in the study are included in the article/supplementary material. Further inquiries can be directed to the corresponding authors.
